# Effectiveness of personal protective health behaviour against COVID-19

**DOI:** 10.1186/s12889-021-10680-5

**Published:** 2021-04-29

**Authors:** Chon Fu Lio, Hou Hon Cheong, Chin Ion Lei, Iek Long Lo, Lan Yao, Chong Lam, Iek Hou Leong

**Affiliations:** 1 Macao Academy of Medicine, Health Bureau, Macao SAR, China; 2 Department of Internal Medicine, Centro Hospitalar Conde de São Januário, Health Bureau, 2nd Floor, Health Bureau Admin, Building, No.339 Rua Nova a Guia, Macao SAR, China; 3 Serviços de Saúde, Edifício da Administração dos Serviços de Saúde, Rua Nova à Guia, n.° 339, Macau SAR, China; 4 Health Bureau, Macao SAR, China; 5 Center for Disease Control and Prevention, Health Bureau, Macao SAR, China

**Keywords:** COVID-19, SARS-CoV-2, Prevention, Measures, Mask, Handwashing, Gathering, Behaviour, Social distancing, Hygiene

## Abstract

**Background:**

Novel coronavirus disease 2019 (COVID-19) has become a pandemic, and over 80 million cases and over 1.8 million deaths were reported in 2020. This highly contagious virus is spread primarily via respiratory droplets from face-to-face contact and contaminated surfaces as well as potential aerosol spread. Over half of transmissions occur from presymptomatic and asymptomatic carriers. Although several vaccines are currently available for emergency use, there are uncertainties regarding the duration of protection and the efficacy of preventing asymptomatic spread. Thus, personal protective health behaviour and measures against COVID-19 are still widely recommended after immunization. This study aimed to clarify the efficacy of these measures, and the results may provide valuable guidance to policymakers to educate the general public about how to reduce the individual-level risk of COVID-19 infection.

**Methods:**

This case-control study enrolled 24 laboratory-confirmed COVID-19 patients from Centro Hospitalar Conde de São Januário (C.H.C.S.J.), which was the only hospital designated to manage COVID-19 patients in Macao SAR, China, and 1113 control participants who completed a 14-day mandatory quarantine in 12 designated hotels due to returning from high-risk countries between 17 March and 15 April 2020. A questionnaire was developed to extract demographic information, contact history, and personal health behaviour.

**Results:**

Participants primarily came from the United Kingdom (33.2%), followed by the United States (10.5%) and Portugal (10.2%). Independent factors for COVID-19 infection were having physical contact with confirmed/suspected COVID-19 patients (adjusted OR, 12.108 [95% CI, 3.380–43.376], *P* < 0.005), participating in high-risk gathering activities (adjusted OR, 1.129 [95% CI, 1.048–1.216], *P* < 0.005), handwashing after outdoor activity (adjusted OR, 0.021 [95% CI, 0.003–0.134], *P* < 0.005), handwashing before touching the mouth and nose area (adjusted OR, 0.303 [95% CI, 0.114–0.808], *P* < 0.05), and wearing a mask whenever outdoors (adjusted OR, 0.307 [95% CI, 0.109–0.867], *P* < 0.05). The daily count of handwashing remained similar between groups. Only 31.6% of participants had a sufficient 20-s handwashing duration.

**Conclusions:**

Participating in high-risk gatherings, wearing a mask whenever outdoors, and practising hand hygiene at key times should be advocated to the public to mitigate COVID-19 infection.

**Supplementary Information:**

The online version contains supplementary material available at 10.1186/s12889-021-10680-5.

## Background

Coronavirus disease 2019 (COVID-19) evolved from a global public health emergency to a pandemic after the declaration by the World Health Organization (WHO) on March 11, 2020 [1]. The outbreak caused public fear and serious burdens on healthcare systems, social relationships and economies worldwide. As of January 3, 2021, over 80 million cases had been confirmed, with more than 1.8 million deaths globally [2]. Nevertheless, the high transmissibility from pre- or asymptomatic patients concurred with virus RNA levels peaking at day 4 from symptom onset, possibly exacerbating the spread [3]. This highly contagious virus is spread primarily via respiratory droplets from face-to-face contact and contaminated surfaces as well as potential aerosol spread [4]. It is estimated that over half of transmissions occur from presymptomatic and asymptomatic carriers [5]. Although vaccines for COVID-19 have become available recently, there are uncertainties regarding the duration of protection and the efficacy of preventing asymptomatic spread [6].

Initially, various public health policies were adopted among different countries to attempt to mitigate the outbreak, and the preliminary outcomes of these measures including “lockdown” or sanitary cordon, travel restrictions, quarantines for travellers, stay-at-home orders, closure of schools and business, and bans on gatherings were encouraging [7–11]. It is recognized that some leading causes of morbidity and mortality could be attributed to health determinants associated with the health behaviour of individuals, such as the adoption of health behaviour against virus transmission (e.g., hand washing and use of masks outdoors) and the avoidance of health-harming behaviours (e.g., touching the face and gathering for occasions) [12]. Though personal hygiene practices such as washing hands, wearing masks, and maintaining social distance are widely recommended to the public based on the knowledge of droplet transmission, there is still scarce evidence of the effectiveness of these personal measures in preventing COVID-19 infection at the individual level.

Therefore, a case-control study was conducted to determine the risk and protective factors for COVID-19 infection at the individual level, with a specific emphasis on personal behaviours such as mask use, the number of gatherings, and hand hygiene practices. A questionnaire was designed to extract related information among COVID-19 patients in Centro Hospitalar Conde de São Januário (C.H.C.S.J.), the only hospital designated to manage COVID-19 patients in Macao SAR. People who had been in a COVID-19 high-risk foreign country undergoing a 14-day mandatory quarantine in 12 designated hotels in Macao SAR served as the control group. This article is structurally divided into the introduction, methods describing the study design and statistical analysis, the results of effect sizes of different measures against COVID-19 infection, discussion and conclusions.

## Methods

### Study design and population

**Fig. 1:**
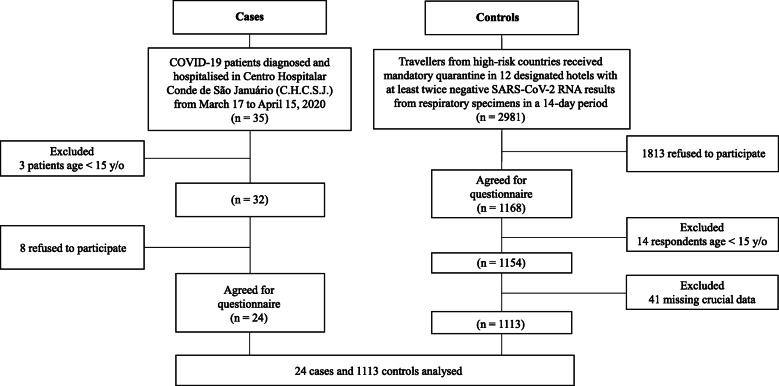
Flowchart of participant recruitment in the case-control study

A cross-sectional questionnaire survey was conducted in Macao from March 17, 2020, to April 15, 2020 (the flowchart of participant recruitment in the case-control study is shown in Fig. 1). The study population consisted of the following: 1) people who had been in a COVID-19 high-risk foreign country in the past 14 days before entry to Macao and would have completed a 14-day mandatory quarantine in 12 designated hotels in Macao before the end of the study period and 2) people diagnosed with COVID-19 and hospitalized in C.H.C.S.J., the only hospital designated to manage COVID-19 cases. All participants who could understand and complete the questionnaire written in Chinese, English or Portuguese were included.

### Instrument and data collection

For the case group, 35 patients were included, 3 patients under the age of 15 were excluded, and 6 patients refused to participate. For the control group, 2981 travellers were initially included, 1813 of whom refused to participate, while 14 respondents under the age of 15 and 41 questionnaires without crucial data were subsequently excluded. The exclusion criteria included age less than 15 years, refusal to participate and questionnaires with missing crucial data. Questions covered the following topics: personal data and information, personal health status and living habits, epidemic prevention and control situation of the main country in which one stayed, contact history, personal protective health behaviour and measures before returning to Macao. The detailed content can be found in the questionnaire template in the Additional file 1.

### Informed consent

The Macao Health Bureau, Centro Hospitalar Conde de São Januário, Medical Ethical Committee approved the research protocol. All procedures performed in the study involving human participants were consistent with the 1964 Declaration of Helsinki and its later amendments or comparable ethical standards. All participants gave informed consent via web-based systems before answering questionnaires. Using a paper questionnaire, written informed consent was acquired and collected.

### Statistical analysis

Descriptive statistics for demographic information, preventive policy, contact history, and personal protective health behaviour and measures were calculated. Then, patients were divided into “COVID-19 infected” and “non-infected” groups. Differences in percentages between groups were examined using Pearson’s chi-square test or Fisher’s exact test. Student’s t-test or Mann–Whitney U test was utilized to examine the differences among continuous variables depending on the data normality. Univariate logistic regression was used to identify factors associated with COVID-19 infection. Then, those significant factors were pooled and selected to build a multivariate logistic model via a forward-selection stepwise method. The level of statistical significance was set at α = 0.05. R (version 3.5.2, R Development Core Team 2018) was used to conduct statistical analyses.

## Results

Overall, 1137 questionnaires were considered effective and were analysed accordingly. The total response rate was 37.7% (1137/3013), and the response rate of the infected group was 75% (24/32). Demographic information and the comparison between infected and non-infected groups are summarized in Table 1. The majority of the participants were aged between 20 and 44 years (65.5%) and had received secondary education or above (55.5%). Overall, 93% of participants denied having any chronic diseases. The most common comorbid diseases were hypertension (3.3%), followed by diabetes mellitus (1.1%) and dyslipidaemia (0.9%). The majority of respondents were non-smokers (80.7%). The top 10 countries in which the participants stayed before returning to Macao were the United Kingdom (33.2%), United States (10.5%), Portugal (10.2%), Australia (9.1%), Canada (4.7%), Philippines (3.5%), China (3.3%), Malaysia (2.4%), Cambodia (2.2%), and Thailand (2%). The main reasons for staying abroad were “study abroad” (60.9%), “visiting relatives” (12.9%), “travel” (11.6%), and “business trip” (6.0%).

**Table 1:** Comparison of demographic information between COVID-19 infected and non-infected participants

	Total (*N* = 1137)	Non-infected (*N* = 1113)	Infected (*N* = 24)	*P* value
**Sex (Male%)**	505/1137 (44.42%)	492/1113 (44.2%)	13/24 (54.17%)	0.331
**Age (mean ± SD)**	28.85 ± 13.23	28.8 ± 13.2	29.8 ± 13.1	0.712
15–19	215/1137 (18.91%)	209/1113 (18.78%)	6/24 (25%)	0.441
20–44	745/1137 (65.52%)	734/1113 (65.95%)	11/24 (45.83%)	0.040
45–54	94/1137 (8.27%)	91/1113 (8.18%)	3/24 (12.5%)	0.442
55–64	54/1137 (4.75%)	53/1113 (4.76%)	1/24 (4.17%)	1.000
65–74	23/1137 (2.02%)	23/1113 (2.07%)	0/24 (0%)	1.000
75–84	3/1137 (0.26%)	3/1113 (0.27%)	0/24 (0%)	1.000
**Education level (%)**				
Primary education	70/1137 (6.16%)	67/1113 (6.02%)	3/24 (12.5%)	0.180
Secondary education	631/1137 (55.5%)	620/1113 (55.71%)	11/24 (45.83%)	0.336
Bachelor’s degree	335/1137 (29.46%)	328/1113 (29.47%)	7/24 (29.17%)	0.974
Master’s degree or above	101/1137 (8.88%)	98/1113 (8.81%)	3/24 (12.5%)	0.465
**Chronic diseases (%)**				
Hypertension	38/1137 (3.34%)	37/1113 (3.32%)	1/24 (4.17%)	0.561
Diabetes mellitus	13/1137 (1.14%)	13/1113 (1.17%)	0/24 (0%)	1.000
Dyslipidaemia	10/1137 (0.88%)	10/1113 (0.9%)	0/24 (0%)	1.000
Gout/hyperuricaemia	7/1137 (0.62%)	7/1113 (0.63%)	0/24 (0%)	1.000
Coronary artery disease	6/1137 (0.53%)	6/1113 (0.54%)	0/24 (0%)	1.000
Hepatitis	6/1137 (0.53%)	5/1113 (0.45%)	1/24 (4.17%)	0.120
Other	36/1137 (3.17%)	35/1113 (3.14%)	1/24 (4.17%)	0.542
**Current smoker (%)**	132/1137 (11.61%)	130/1113 (11.68%)	2/24 (8.33%)	1.000
Ex-smoker (%)	87/1137 (7.65%)	84/1113 (7.55%)	3/24 (12.5%)	0.422
Never-smoker (%)	918/1137 (80.74%)	899/1113 (80.77%)	19/24 (79.17%)	0.844
**Alcohol consumption (%)**	37/1137 (3.25%)	36/1113 (3.23%)	1/24 (4.17%)	0.552

### Personal protective health behaviour and measures for COVID-19 in the local community in which the participants stayed before returning to Macao

Within 14 days before returning to Macao, the majority of respondents (79.3%) stated that COVID-19 was spreading in the countries in which they stayed (Table 2). In total, 42.9% of participants were requested to undergo self-quarantine at home. There were seemingly lower proportions of traffic restrictions (20.8% vs 33.2%; *P* = 0.201) and closures of public entertainment venues (37.5% vs 49.2%; *P* = 0.255) in the COVID-19-infected group than in the non-infected group in the countries in which they stayed; however, the statistical power was insufficient to differentiate the extent of these differences due to the limited sample size in the COVID-19 patient group.

**Table 2:** Preventive measures for COVID-19 in communities where participants stayed before returning to Macao in 2020

	Total (*N* = 1137)	Non-infected (*N* = 1113)	Infected (*N* = 24)	*P* value
**Within 14 days before returned to Macao**				
COVID-19 was spreading in the country in which you stayed (Yes, %)	902/1137 (79.33%)	884/1113 (79.42%)	18/24 (75%)	0.596
Underwent mandatory self-quarantine at home (Yes, %)	488/1137 (42.92%)	477/1113 (42.86%)	11/24 (45.83%)	0.771
Had traffic restrictions in the country in which you stayed (Yes, %)	375/1137 (32.98%)	370/1113 (33.24%)	5/24 (20.83%)	0.201
Provided COVID-19 testing for every symptomatic people in the country in which you stayed (Yes, %)	642/1137 (56.46%)	632/1113 (56.78%)	10/24 (41.67%)	0.139
Public entertainment venues were shut down in the country in which you stayed (Yes, %)	557/1137 (48.99%)	548/1113 (49.24%)	9/24 (37.5%)	0.255

### Contact history and frequency of outdoor activities

In total, only a minority of participants had visited medical facilities for any reason (4.9%) and had contact with those who had respiratory symptoms (8%) or confirmed/suspected COVID-19 patients (2.2%) (Table 3). Notably, there were significantly higher percentages of these activities in the infected group than in the non-infected group, such as having physical contact with those who had respiratory symptoms (25% vs 7.6%; *P* = 0.002) or confirmed/suspected COVID-19 patients (16.7% vs 1.9%; *P* = 0.001). Moreover, compared to the non-infected populations, the infected group presented fewer protective measures during/after contact with high-risk people, such as washing hands (50% vs 95.3%; *P* = 0.005) and wearing a mask (16.7% vs 67.1%; *P* = 0.022) following contact with someone who was symptomatic. Participants were asked to calculate the total number of outdoor activities within a 14-day interval before returning to Macao. Notably, there were significantly more high-risk gathering activities defined by interacting with people within 2 m without wearing a mask (5.4 ± 10.1 vs 0.7 ± 2.3; *P* = 0.034) in the infected group than in the non-infected group within 14 days before returning to Macao.

**Table 3:** Contact history and frequency of outdoor activities among participants 14 days before returning to Macao

	Total (*N* = 1137)	Non-infected (*N* = 1113)	Infected (*N* = 24)	*P* value
**Contact history**				
Went to any hospitals or clinics for any reason (Yes, %)	56/1137 (4.93%)	53/1113 (4.76%)	3/24 (12.5%)	0.110
Went to hospitals or clinics for respiratory symptoms (Yes, %)	20/1137 (1.76%)	18/1113 (1.62%)	2/24 (8.33%)	0.064
Had physical contact with anyone who had respiratory symptoms (Yes, %)	91/1137 (8%)	85/1113 (7.64%)	6/24 (25%)	0.002
If yes, did you wear a mask during your contact (Yes, %)	58/91 (63.74%)	57/85 (67.06%)	1/6 (16.67%)	0.022
If yes, did you wash your hands after your contact (Yes, %)	84/91 (92.31%)	81/85 (95.29%)	3/6 (50%)	0.005
Had physical contact with suspected/confirmed COVID-19 patients (including family members) (Yes, %)	25/1137 (2.2%)	21/1113 (1.89%)	4/24 (16.67%)	0.001
If yes, did you wear a mask during the contact (Yes, %)	13/25 (52%)	13/21 (61.9%)	0/4 (0%)	0.039
If yes, did you wash your hands after your contact (Yes, %)	25/1137 (2.2%)	20/21 (95.24%)	2/4 (50%)	0.057
**Frequency of outdoor activities** **(Total number measured in a 14-day interval before returning to Macau)**				
Went to workplace (mean ± std. deviation)	0.73 ± 2.41	0.7 ± 2.4	0.8 ± 2.6	0.833
Went to school (mean ± std. deviation)	2.32 ± 3.7	2.3 ± 3.7	3.4 ± 5.1	0.291
Went to crowded places, such as supermarkets, malls, and cinemas (mean ± std. deviation)	2.34 ± 2.43	2.3 ± 2.4	3.8 ± 4.6	0.142
Took public transportation vehicles, such as bus, underground transit, train, and aircraft but excluding taxi (mean ± std. deviation)	2.52 ± 4.36	2.5 ± 4.3	4.5 ± 5.7	0.110
Participated in high-risk gathering activities defined by interacting with people within 2 m without wearing a mask such as parties, bars, restaurants, family and friend gatherings (mean ± std. deviation)	0.82 ± 2.77	0.7 ± 2.3	5.4 ± 10.1	0.034

### Mask usage behaviour, timing and duration for handwashing

More than half (63.5%) of the participants admitted that they wore a mask whenever they stayed outdoors within 14 days before returning to Macao. There was a larger proportion of non-infected participants wearing a mask whenever outdoors than the infected group (63.5% vs 25.0%; *P* < 0.001) (Table 4). The majority of participants believed that there was a lower chance of accidentally touching the mouth and nose area after wearing a mask (79.8%), and almost all of them acknowledged that hand hygiene was still important after mask usage (95.1%).

**Table 4:** Mask usage behaviour, timing and duration for handwashing among participants

	Total (*N* = 1137)	Non-infected (*N* = 1113)	Infected (*N* = 24)	*P* value
**Within 14 days before you returned to Macao, did you wear a mask when outdoors?**				
Each time (%)	713/1137 (62.71%)	707/1113 (63.52%)	6/24 (25%)	< 0.001
Sometimes (%)	248/1137 (21.81%)	242/1113 (21.74%)	6/24 (25%)	0.702
Seldom (%)	91/1137 (8%)	88/1113 (7.91%)	3/24 (12.5%)	0.412
Never (%)	84/1137 (7.39%)	76/1113 (6.83%)	8/24 (33.33%)	< 0.001
**Your opinion on the frequency of accidentally touching the mouth and nose area after wearing a mask**				
Less (%)	906/1134 (79.89%)	888/1113 (79.78%)	18/21 (85.71%)	0.783
Same (%)	178/1134 (15.7%)	177/1113 (15.9%)	1/21 (4.76%)	0.230
Increase (%)	50/1134 (4.41%)	48/1113 (4.31%)	2/21 (9.52%)	0.236
Do you think that hand hygiene is less important after wearing a mask? (yes, %)	56/1134 (4.94%)	55/1113 (4.94%)	1/21 (4.76%)	1.000
**Did you frequently wash hands under the following situations within 14 days before returning to Macao?**				
When your hands are visibly dirty (Yes, %)	906/1137 (79.68%)	885/1113 (79.51%)	21/24 (87.5%)	0.447
Before eating (Yes, %)	832/1137 (73.18%)	818/1113 (73.5%)	14/24 (58.33%)	0.087
Before handling food or cooking (Yes, %)	1041/1137 (91.56%)	1020/1113 (91.64%)	21/24 (87.5%)	0.448
After handling food or cooking (Yes, %)	1066/1137 (93.76%)	1048/1113 (94.16%)	18/24 (75%)	< 0.001
After defecation (Yes, %)	1084/1137 (95.34%)	1063/1113 (95.51%)	21/24 (87.5%)	0.097
After a toilet trip (Yes, %)	1037/1137 (91.2%)	1018/1113 (91.46%)	19/24 (79.17%)	0.035
After outdoor activity (Yes, %)	1127/1137 (99.12%)	1107/1113 (99.46%)	20/24 (83.33%)	< 0.001
Before attending to a child or sick person (Yes, %)	1048/1137 (92.17%)	1028/1113 (92.36%)	20/24 (83.33%)	0.112
After attending to a child or sick person (Yes, %)	1047/1137 (92.08%)	1027/1113 (92.27%)	20/24 (83.33%)	0.115
After sneezing or coughing (Yes, %)	909/1137 (79.95%)	896/1113 (80.5%)	13/24 (54.17%)	0.001
After handling pet (Yes, %)	918/1137 (80.74%)	904/1113 (81.22%)	14/24 (58.33%)	0.005
Before touching the mouth and nose area (Yes, %)	975/1137 (85.75%)	963/1113 (86.52%)	12/24 (50%)	< 0.001
**Duration for handwashing each time within 14 days before returning to Macao (seconds, mean ± std. deviation)**	23.6 ± 16.1	23.7 ± 16.1	18.8 ± 11.2	0.136
**Handwashing for over 20 s each time (yes, %)**	356/1127 (31.59%)	352/1103 (31.91%)	4/24 (16.67%)	0.125
**The estimated number of handwashes with soap or alcoholic sanitizers per day within 14 days before returning to Macao (mean ± std. deviation)**	9.2 ± 8.4	9.2 ± 8.4	9.1 ± 8.4	0.958

With regard to hand hygiene, the practice of handwashing was substantially less common in the infected population than in the non-infected population, such as handwashing after handling food or cooking (75% vs 94.2%; *P* < 0.001), after a toilet trip (79.2% vs 91.5%; *P* = 0.035), after outdoor activity (83.3% vs 99.5%, *P* < 0.001), after sneezing or coughing (54.2% vs 80.5%; *P* = 0.001), after handling pets (58.3% vs 81.2%; *P* = 0.005), and before touching the mouth and nose area (50.0% vs 86.5%; *P* < 0.001). However, only approximately one-third of the total population (31.6%) achieved a sufficient 20-s duration for handwashing. Furthermore, only 16.7% of the infected population washed hands for over 20 s each time, compared with 31.9% in the noninfected group (*P* = 0.125), and the mean duration of handwashing each time was less than 20 s in the infected group (18.8 ± 11.2 s). On the other hand, the average number of handwashes with soap or alcoholic sanitizers per day remained similar between the two groups (9.1 ± 8.4 vs 9.2 ± 8.4, *P* = 0.958).

### Risk and protective factors associated with COVID-19 infection

In univariate analysis (Table 5), those who had physical contact with people having respiratory symptoms (crude OR, 10.4 [95% CI, 3.270–33.079], *P* < 0.005) or confirmed/suspected COVID-19 patients (crude OR, 12.381 [95% CI, 4.261–35.973], *P* < 0.005) had a higher risk of COVID-19 infection than those who did not. A risk reduction of 80.9% was noted in those who wore masks whenever outdoors (crude OR, 0.191 [95% CI, 0.075–0.486], *P* < 0.005) compared with those who did not. Outdoor activities, such as “high-risk gathering”, defined as interacting with people within 2 m without wearing masks, significantly increased the COVID-19 risk by up to 15.5% each time (crude OR, 1.155 [95% CI, 1.089–1.225], *P* < 0.005). Decent handwashing habits showed protective effects on COVID-19 infection, such as after handling food or cooking (crude OR, 0.186 [95% CI, 0.071–0.485], *P* < 0.005), after a toilet trip (crude OR, 0.355 [95% CI, 0.130–0.971], *P* < 0.05), after outdoor activity (crude OR, 0.027 [95% CI, 0.007–0.104], *P* < 0.005), after sneezing or coughing (crude OR, 0.286 [95% CI, 0.127–0.648], *P* < 0.005), after handling pets (crude OR, 0.324 [95% CI, 0.142–0.739], *P* < 0.01), and before touching the mouth and nose area (crude OR, 0.156 [95% CI, 0.069–0.353], *P* < 0.005). In multivariate logistic regression via a forward-selection stepwise method, independent factors for COVID-19 infection were having physical contact with confirmed/suspected COVID-19 patients (adjusted OR, 12.108 [95% CI, 3.380–43.376], *P* < 0.005), wearing a mask whenever outdoors (adjusted OR, 0.307 [95% CI, 0.109–0.867], *P* < 0.05), the number of high-risk gathering activities (interact with people within 2 m without wearing a mask) in a 14-day interval (adjusted OR, 1.129 [95% CI, 1.048–1.216], *P* < 0.005), handwashing after outdoor activity (adjusted OR, 0.021 [95% CI, 0.003–0.134], *P* < 0.005), and before touching the mouth and nose area (adjusted OR, 0.303 [95% CI, 0.114–0.808], *P* < 0.05).

**Table 5:** Univariate and multivariate logistic regression to assess the effectiveness of protective health behaviour and measures

	Crude Odds Ratio (95% CI)	Adjusted Odds Ratio^a^ (95% CI)
Age	1.005 (0.977, 1.035)	
Male	1.492 (0.663, 3.359)	
Hypertension	1.163 (0.153, 8.817)	
Had physical contact with anyone who had respiratory symptoms	10.4 (3.270, 33.079)***	
Had physical contact with suspected/confirmed COVID-19 patients	12.381 (4.261, 35.973)***	12.108 (3.380, 43.376)***
Wearing a mask whenever outdoors	0.191 (0.075, 0.486)***	0.307 (0.109, 0.867)*
Participated in high-risk gathering activities (interacted with people within 2 m without wearing a mask)	1.155 (1.089, 1.225)***	1.129 (1.048, 1.216)***
Wash hands after handling food or cooking	0.186 (0.071, 0.485)***	
Wash hands after a toilet trip	0.355 (0.130, 0.971)*	
Wash hands after outdoor activity	0.027 (0.007, 0.104)***	0.021 (0.003, 0.134)***
Wash hands after sneezing or coughing	0.286 (0.127, 0.648)***	
Wash hands after handling pets	0.324 (0.142, 0.739)**	
Wash hands before touching the mouth and nose area	0.156 (0.069, 0.353)***	0.303 (0.114, 0.808)*
Handwashing for over 20 s each time	0.427 (0.145, 1.258)	

^a^ Univariate logistic regression was used to identify factors associated with COVID-19 infection. Then, those significant factors were pooled and selected to build a multivariate logistic model via a forward-selection stepwise method

*P < 0.05

**P < 0.01

***P < 0.005

## Discussion

Ultimately, our findings showed that the most commonly advised measures were effective against COVID-19 infection. Most of the participants in both the infected and non-infected groups were healthy, young students studying abroad. The infected group consisted of large numbers of people returning from the United Kingdom and the Philippines. This factor somehow correlated with these countries’ community outbreaks during that period. Moreover, COVID-19 patients responded that there were fewer public preventive measures taken in the local community where they stayed before returning to Macao SAR, such as closing entertainment venues, traffic restrictions, and testing COVID-19 for all symptomatic patients. From Wuhan’s report in China, suspending intracity public transport, closing entertainment venues, and banning public gatherings were associated with reductions in case incidence [13]. Although the statistical power was insufficient to distinguish differences in public measures among the uninfected and infected groups in this study, our data showed that each high-risk gathering activity (interacting with people within 2 m without wearing a mask) increased the risk of COVID-19 infection by 12.9%.

Significant exposure to COVID-19 was commonly defined as face-to-face contact within 6 ft (~ 1.83 m) with symptomatic COVID-19 patients that was sustained for at least a few minutes [14]. The transmission of viruses was reported to be lower with physical distancing of 1 m or more, for which protection would be increased with increasing distance [15]. Based on our data, physical contact with suspected/confirmed COVID-19 cases increased the risk of infection by 12-fold. While this contact history may be retrospective in nature, this finding revealed the differences in personal hygiene behaviour between the control group and the COVID-19 infection group when they had contact with symptomatic individuals. In the infection group, 50.4% fewer people wore a mask when contacting people with respiratory symptoms, and 45.3% fewer people washed hands afterwards. As a result, we believe that personal protective health behaviour such as hand hygiene, especially after high-risk activities, and mask-wearing could be crucial to prevent transmission from highly contagious individuals. However, in this study, the small sample size of patients with definite contact history limited the calculation of the actual effect size of these protective measures.

The WHO stated that the use of a mask alone is insufficient to provide an adequate level of protection and that other measures such as hand hygiene should also be adopted to prevent human-to-human transmission of COVID-19 [16]. Traditionally, the role of wearing a mask by a healthy citizen has been controversial, and the limited evidence has mostly been associated with a healthcare setting [17, 18]. From a systematic review and meta-analysis, face mask use could result in a large reduction in the risk of reduction (pooled adjusted odds ratio 0.18) [15]. Our data showed similar evidence in that outdoor mask wearing in healthy populations reduced COVID-19 risk by 69.3% after adjusting for contact history, hand hygiene practice, and high-risk gathering activities. However, the questionnaire in this study did not specify the type of face mask worn. Although incorrect use of masks may lead to virus colonization and self-contamination, [19] 79.9% of our participants thought that mask wearing reduced the frequency of accidentally touching the mouth and nose area, with 95.1% of them recognizing the importance of hand hygiene after using a mask. This result indicated that most of the participants had a good perceived hygiene attitude on mask usage; hence, the protective effect might exceed the potential risk in this circumstance. We believe that mask wearing by “non-sick” people could potentially block the spread of contagious droplets from asymptomatic patients during social activities as well as provide the wearer with a symbol to enhance the awareness of protective measures and generate a sense of safety and well-being. Nonetheless, multiple factors should be taken into consideration before implementing a universal mask policy in a healthy population, including cultural differences, scientific evidence in different settings, adequacy of perceived knowledge on mask use in the general population, adaptation difficulties in people with special needs and, most importantly, the scarcity of resources and logistic support [20–23].

Previous studies mostly aimed to evaluate the protective efficacy of physical distancing, wearing masks, eye protection, etc., in both healthcare and non-healthcare settings [15, 24, 25]. However, there is still scarce evidence regarding hand hygiene in preventing individual COVID-19 infections. Hand hygiene is regarded as one of the most effective measures for transmissible disease prevention [18, 26]. Special emphases were placed on the timing and duration of cleaning in this study. Our data suggested that the most important protective factor for COVID-19 was the timing of hand hygiene practice and not the frequency. The habit of frequent handwashing after outdoor activities and before touching the mouth/nose area reduced the risk of infection by 97.9 and 69.7%, respectively. There is evidence that viruses can remain viable and infectious on surfaces for up to days, leading to plausible fomite transmission [27]. Transmission of the virus via contaminated surfaces was also proven to be a possible means other than via respiratory droplets from face-to-face contact [28, 29]. These results reiterate the importance of practising hand hygiene following outdoor activities, even if no obvious high-risk contact was noted, as the battlefield is limited not only to healthcare settings but also to asymptomatic or presymptomatic carriers in public places [4]. Nevertheless, the duration of handwashing was seemingly shorter in the infected group than in the non-infected group, although the difference was not statistically significant. The primary challenges associated with hand hygiene efficacy are the laxity of practice and atopic dermatitis [30, 31]. A cross-sectional survey of the general public reported that only approximately 31% of the expected behaviour and practices were observed in at least 80% of the participants [32]. Our data showed that the percentages of each hand hygiene behaviour were higher than those reported previously [32]. This fact may contribute to awareness of the COVID-19 pandemic and active advocation and education to the public by different authorities and organizations. During the 2003 SARS outbreak, compliance with hand hygiene practice was also improved among medical students [33].

This study had several limitations. First, this was a retrospective survey, and recall bias was inevitable. However, the effect of bias was minimized since the information requested was about simple concrete behaviour and events that happened recently, and the associations had strong effect sizes. Second, the sample size of the infected group was relatively small compared to that of the non-infected group, which was limited due to the unavailability of confirmed cases. In addition, the low response rate in the control group may have been a consequence of implementing an internet-based questionnaire. Future studies may consider using reminders to boost the response rate. Third, the lack of objective evaluation of behaviour and practice may not reflect the consistency between attitude and actual behaviour. Furthermore, the results may be limited to the Asian population during the COVID-19 outbreak, and generalization of these interpretations to other populations should be thoughtfully considered.

## Conclusions

Our data provide evidence of the effectiveness of personal protective measures against COVID-19 infection. Although vaccines are now available for emergency use in many countries, there is some uncertainty around the efficacy of stopping asymptomatic spread via vaccination. It is not unreasonable that policymakers continue to educate the public about avoiding high-risk gatherings, wearing a mask whenever outdoors, and practising decent hand hygiene along with the immunization scheme. Based on the relatively small sample size in our patient group, future studies may recruit more participants to validate the effectiveness of these measures in different populations.

## Supplementary Information

**Additional file 1.**

## Data Availability

The dataset supporting the conclusions of this article is included within the article and its additional file [see Additional file 1].

## Abbreviations

COVID-19: Novel coronavirus disease 2019; WHO: World Health Organization
C.H.C.S.J.: Centro Hospitalar Conde de São Januário
