# Supplementary material for: Effectiveness of personal protective health behaviour against COVID-19
Source: BMC Public Health. 2021 Apr 29;21:827. doi: 10.1186/s12889-021-10680-5 (PMC8082215; doi:10.1186/s12889-021-10680-5)
Supplement: Supplementary file 1 — Additional file 1. [file 12889_2021_10680_MOESM1_ESM.docx]

**Questionnaire research for people returning Macao from other countries**

| Dear Sir/Madam,  In order to understand your protective measures, individual health status and living habits under the Coronavirus disease 2019 (COVID-19) pandemic, this information is a high valuable referential data for this bureau on developing protection and control measures against new NCP epidemic situations.  You are kindly requested to take a few minutes to complete the following questionnaire, so please fill in the answer according to your own situation.  The survey’s collected information will be kept anonymous. Your personal data only will be used by the government as a reference for epidemic preventive policies.  Sincere gratitude on your participation, cooperation and support! Thank you for your concerted efforts in combating the epidemic!   - Agree   □ Don’t agree (Thanks for supporting!) |
| --- |

1. **Personal data**

Gender ：□ Male □ Female Date of birth：

Nationality： 　 　　　　　Occupation：

- Macao BIR No.：
- Other Identity No.：　　　　　 　　 Education level：

□ 小學畢業 Primary school degree

□ 中學畢業 Secondary school degree

□ 大學學士畢業 Bachelor’s degree

□ 碩士或以上畢業 Master degree or above

- Main Country you stayed before return to Macao：
- Main reason for visit / stay (Single option) :

□ Travel

□ Visit relative

□ Study abroad

□Official mission / Business

□Others, please list out ：

- Number of people co-habit in the living place of the main country you stayed ：

　　　　　　　　 people

- Area of the living place / household in the main country you stayed ：

　　　　　　　　　 square feet^2^ /meter^2^

- Main reason of returning to Macao at this time (Single option)

□Epidemic spread of COVID-19

□Worried about insufficiency of medical support abroad

□Worried about continue of the epidemic abroad may lead to impossibility of returning to Macao within a short period of time

□Missing your family

□Others, please list out：

1. **Personal health status and living habits**
2. Do you have any chronic disease?
   󠄁 No

󠄁 If Yes, please tick the illness(es) you have:

□Hypertension

□ Cardiac / heart disease

□ Diabetes

□ Hyperlipidemia

□ Gout/ High urine acid

□Cerebral vascular disease (Stroke)

□Chronic renal disease

□Hepatitis

□Cirrhosis

□Chronic Obstructive Pulmonary Disease (COPD)

□Others, please list out：

1. Do you have smoking habit?󠄁

□Yes □Already ceased smoking □Never

1. Do you have alcoholic drinking habit?󠄁

□Yes □No

1. Your level of anxiety during your stay in the main country abroad

(1 Point: No worry at all; 10 Point: Very anxious)

󠄁１□２□３□４□５□６□７□８□９□１０

1. During your stay in the main country abroad, if you felt anxious, what were the sources of your worries? (Can choose more than one option)

□ Epidemic abroad is expanding

□ Worried about insufficiency of medical support abroad

□Worried about continue of the epidemic abroad may lead to impossibility of returning to Macao within a short period of time

□Missing your family

□Feeling alone

□Others, please list out _____________

1. Your level of anxiety after you returned to Macao (1 point: No worry at all; 10 points: Very worried)
   󠄁１□２□３□４□５□６□７□８□９□１０
2. After you returned to Macao, if you have felt anxious, what is the source of your anxiety? (Can choose more than one)

□Medical observation

□Got infection during the travel

□Unable to attend school / work in that country

□Others, please list out

1. After you returned to Macao, what is the reason of mood improvement if you have?

1. **Epidemic prevention and control situation of the main country you stayed**
   1. Within 14 days before you returned to Macao, the epidemic was expanding in the country you have stayed？　　　　　　　　 　 □Yes　　 □No
   2. Within 14 days before you returned to Macao, was there any mandatory orders of quarantine in the country you have stayed？　　　　　　 □Yes　　□No
   3. Within 14 days before you returned to Macao, was there any traffic restrictions in the country you have stayed？ □ Yes　　□No
   4. Within 14 days before you returned to Macao, was there any COVID-19 tests for symptomatic people in the country you stayed?　　 □Yes　　□No
   5. Within 14 days before you returned to Macao, were the public entertainment spots closed in the country you stayed？　　　　 □Yes　　□No
   6. Within 14 days before you returned to Macao, were the supermarkets opened as usual？

□Yes　 □No

- 1. Within 14 days before you returned to Macao, were both masks and hand sanitizers available for sale in the country you stayed？ □Yes　　□No

1. **History of contact**
   1. Within 14 days (2 weeks) before you returned to Macao, did you go to any hospitals or clinics？ □ Yes　　□No

If yes, reasons for going there：□ Visit sick people □ seek for medical consultation □accompany a sick person □work

- 1. Within 14 days before you returned to Macao, did you go to hospitals or clinics for respiratory symptoms (such as: cough)？ □ Yes　　□ No

If yes, did you get tested for SARS-CoV2? □ Yes　　□ No

- 1. Within 14 days before you returned to Macao, did your general medical practitioner / family doctor contact you？ □ Yes　　□ No

If yes, did he/she provide any suggestion/information on the epidemic

- Yes, please specify the suggestion：
- No
  1. Within 14 days (2 weeks) before you returned to Macao, did you have any physical contact with confirmed/suspected patients with COVID-19 (including family members) ？　　　　　　　　　　　　□ Yes □ No

If yes, did you wear mask when you had contact with them 　　　　　　　　　　　　 　□ Yes　　□ No

If yes, did you wash your hands after your contact with them

□Yes　　 □ No

- 1. Within 14 days (2 weeks) before your return to Macao, did you have physical contact with anyone who had respiratory symptoms (such as cough) ？

□ Yes　　 □ No

If yes, did you wear mask during your contact with them 　　　　　　　　　　　　 　　□Yes　　 □ No

If yes, did you wash your hands after your contact with them

□Yes　　 □ No

- 1. Within 14 days (2 weeks) before you returned to Macao, did you have the following actions？If yes, please specify the frequency, if no, please fill in 0
- Went to workplace：14days（2 weeks）Total times
- Went to school：14days（2 weeks） Total times
- Went to crowding places (such as: supermarkets, malls, cinemas) ：14days（2weeks） Total times
- Did you take public transportation vehicles (such as: bus, underground rail, train, aircraft, **excluding taxi**)：

14days（2 weeks）Total times

（Single ride will be counted as once, and “go and return” trip will be counted as twice）

- Participated in high-risk gathering activities (interact with people within 2 meters without wearing mouth mask, such as: parties, bars, restaurants, family and friends gatherings：

14days（2 weeks）Total times

- Difference in frequency of gatherings before the epidemic

□ More 　 □ Same 　□ Less

- 1. During your stay abroad, your personal opinion that the highest risk of getting infection is:

□At work/ at school □Family and friends gatherings

□Public transportation vehicles

□Supermarkets/malls/cinemas

1. **Personal protective measures**
   1. Where do you get the protective care information? (Can choose more than one option)

□ TV □ Radio □ Newspapers □Social media (such as Facebook)

- 1. Education level ：

□ Primary degree □Secondary degree

□Bachelor’s degree □Master degree or above

- 1. Within 14 days before you returned to Macao, did you wear mask when going out? □Each time □Sometimes □Rare □Never
  2. Before the epidemic, if you got influenza, did you wear mask when going out?

□Each time □Sometimes □Rare □Never

- 1. Your opinion on frequency of accidental touching on mouth and nose by hands after you wear mouth mask

□Less □Same 　 □More

- 1. Do you think hands hygiene is less important after mask wearing?

□Yes □No

- 1. Within 14 days before you returned to Macao, what were the number of times on washing hands with soap/liquid soap/alcoholic sanitizers per day?

Total times each day

- 1. Within 14 days before you returned to Macao, did you frequently wash hands under the following situation?

When your hands are visibly dirty? 　　　　 □Yes □No

Before eating?　　　　　　　　　　　　　　　　 □Yes □No

Before handling food or cooking?　　　　　　　　 □Yes □No

After handling food or cooking? 　　　　　　　　 □Yes □No

After defecation? 　　　　　　　　　　　　　 □Yes □No

After a toilet trip? 　　　　　　　 □Yes □No

After an outdoor activity?　　　　　　　　　　 □Yes □No

Before attending to a child or sick person? □Yes □No

After attending to a child or sick person?　 □Yes □No

After sneezing or coughing? 　　　　　 □Yes □No

After handling pets? 　　　　　　 □Yes □No

Before touching mouth and nose □Yes □No

- 1. Within 14 days before you returned to Macao, how long does it take for hands washing each time?

Around 　　　　seconds

- 1. Before the epidemic, what were the number of times on washing hands with soap/liquid soap/alcoholic sanitizers each day?

**Each day** total 　　　　　times

-------------------------------------------------------------------------------------------------------------------

Questionnaire accomplished, thank you for your participation!
